# Improving the Estimation of p*K*_a_ Values for All Titratable Amino Acids at the Water/Membrane Interface

**DOI:** 10.1021/acs.jpcb.5c07807

**Published:** 2026-04-13

**Authors:** Nuno F. B. Oliveira, Pedro B. P. S. Reis, Miguel Machuqueiro

**Affiliations:** † BioISI—Instituto de Biossistemas e Ciências Integrativas, Departamento de Química e Bioquímica, Faculdade de Ciências, 111161 Universidade de Lisboa, 1749-016 Lisboa, Portugal; ‡ Machine Learning Research, 1569 Bayer AG, Müllerstraße 178, 13353 Berlin, Germany

## Abstract

Understanding how pH-sensitive molecules behave when incorporated into lipid bilayers is a challenging problem that requires specialized methods and robust sampling. Techniques based on constant-pH molecular dynamics (CpHMD) can be employed to investigate the effects of pH on molecules at the water-membrane interface. Here, we compare the performance of enhanced sampling protocols coupled with CpHMD in describing protonation and p*K*_a_ values of model Ala pentapeptide systems inserting into a DMPC membrane. From regular CpHMD, replica exchange (pHRE), and umbrella sampling (US-CpHMD), the latter provides more reliable and complete p*K*_a_ profiles along bilayer insertion, in particular, at deeper regions of the membrane. Overall, there is a consistent trend for p*K*_a_ values to shift toward the molecules’ neutral state, a crucial effect that enables a (de)­protonation-assisted increase in membrane permeability. Building on US-CpHMD, we have successfully coupled an umbrella replica exchange scheme (REUS), which significantly improved the reliability of results for our Asp pentapeptide by enhancing configurational sampling between the umbrellas, albeit at a substantial computational cost.

## Graphical abstract

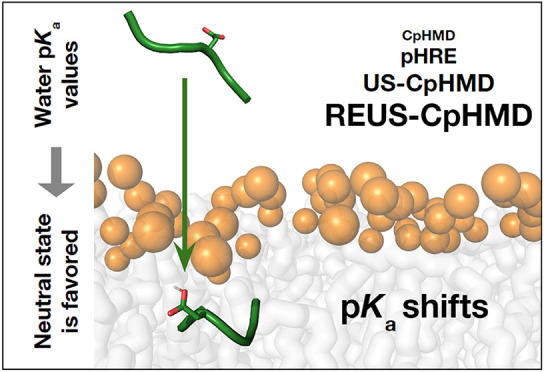

## Introduction

The concentration of hydrogen ions is one of the most important parameters in any solution. In biological systems, the intracellular pH value is tightly regulated, as it influences the structure and function of most biomolecules.^1,2^ This regulation impacts all biochemical processes, including protein folding,^3–5^ lipid bilayer properties,^6–8^ or lipid–protein interactions.^9,10^ The effect of pH on biomolecules arises from the (de)­protonation of titratable sites. The surrounding electrostatic environment influences the protonation equilibrium of each site. In proteins, multiple titratable residues often influence one another’s preferred protonation states.^11^ This complex network of interacting residues can induce and be induced by large conformational transitions that are difficult to predict.^12,13^ The coupling between the conformational space and protonation is well illustrated by the significant shifts observed in the p*K*_a_ values of several amino acids in the unfolding event of a protein.^14,15^ Changes in the solvent mixture can also influence the p*K*_a_ values of titratable amino acids,^16^ as well as insertion into a lipid bilayer.^17–33^

Biological membranes can be highly complex, and the protonation/deprotonation events of molecules interacting with and inserting into membranes are challenging to observe. In some cases, the protonation of several groups is coupled to an event that is more accessible to experimental methods.^17–20^ The Engelman group used tryptophan fluorescence to measure the p*K* value for pHLIP peptide insertion. This is an estimate of the macroscopic p*K*_a_ of at least one pH-sensitive group that titrates at the moment of membrane insertion, when it can still access solvent protons. However, it was very difficult to assign specific p*K*_a_ values to individual groups.^17,19^

Computational methodologies can complement wet lab methodologies, providing valuable insights to understand the pH effect in lipid bilayer systems^22,25,26,34–37^ and its influence in the interaction between peptides and lipidic membranes.^22,25,30,31,38–42^

In a previous work, we have applied the stochastic constant-pH molecular dynamics (CpHMD) method to determine the p*K*_a_ value profile of titratable amino acids in alanine-based pentapeptides^43,44^ (Ala_2_–X–Ala_2_, where X is a titrating residue).^30^ The data showed that fixed protonation states are insufficient to capture the pH effects in membrane-interacting titratable amino acids, since their preferred charge state greatly varies along the membrane normal.^30^ As expected, all residues tend to adopt their neutral state upon insertion into the apolar membrane. However, protonation sampling of the less solvent-exposed peptide conformations was poor, making it difficult to accurately estimate the ratio of ionized to neutral species.

Generalized ensemble methods,^45,46^ such as simulated tempering^47^ and temperature replica exchange molecular dynamics,^48^ are typically employed to overcome kinetic trapping in molecular dynamics (MD) simulations. A temperature replica-exchange scheme was successfully implemented by Khandogin et al.,^49^ thereby improving the p*K*_a_ convergence of their CpHMD method. However, increasing the temperature could alter membrane properties, inducing undesired lipid phase transitions. Alternatively, we have implemented a pH replica exchange in our CpHMD method (pHRE),^50^ similar to the one reported by Roitberg’s group.^51^ In this method, each replica has a unique pH value and exchanges are attempted periodically between pairs of replicas.

Another way to enhance sampling of rare events is to use metadynamics and umbrella sampling.^52,53^ Recently, metadynamics was coupled to the stochastic titration CpHMD to capture nucleotide titration events in single-stranded RNAs.^54^ We have also successfully integrated an umbrella sampling scheme into our CpHMD methodology,^55^ and its application to exploring the protonation/conformation coupling of pentapeptides inserting into a lipid bilayer seems promising. Here, following the strategy introduced in our previous replica-exchange implementation (pHRE),^50^ we propose coupling an umbrella-sampling replica-exchange scheme to CpHMD (REUS-CpHMD). In REUS-CpHMD, conformations from adjacent umbrella windows, at the same pH and temperature, attempt to exchange periodically, with acceptance following the Metropolis criterion. This new st-CpHMD integration will benefit from increased sampling in the US scheme and will also enhance sampling of regions between umbrellas, a known weakness of the parent method for membrane insertion phenomena.^56^

In this work, we study the membrane insertion of Ala-pentapeptides containing pH-titrating residues in the middle position and compare four different sampling techniques, CpHMD simulations,^30^ pHRE,^50^ US-CpHMD,^55^ and the REUS-CpHMD implemented here (Table S1 of the Supporting Information). Although the pHRE and US-CpHMD methods have been previously introduced, this is their first application to enhance the conformational and protonation sampling of titratable peptides partitioning into a membrane bilayer. Here, we also implemented a REUS-CpHMD method, which couples the replica-exchange scheme with the US-CpHMD protocol to yield the sampling benefits of both parent methods. Therefore, we present a comprehensive comparison of methodologies to facilitate the selection of the most suitable computational pipeline for investigating the complexity of pH-dependent membrane insertion processes. The work aims to identify optimal protocols to accurately study the effects of pH on the transport of small molecules across membranes and their impact on drug development.

## Methods

In this work, several CpHMD-based methodologies were employed, some of which are already implemented in our framework. The stochastic constant-pH Molecular Dynamics (CpHMD)^57,58^ method has already been used in a preliminary study involving the same pentapeptide systems interacting with a lipid bilayer.^30^ The replica-exchange CpHMD (pHRE),^50^ and umbrella sampling CpHMD (US-CpHMD)^55^ have been previously developed and implemented, and are readily available in our CpHMD pipeline. In addition to these more common methods, we have coupled the replica-exchange scheme with the US-CpHMD framework, thereby implementing the first double-enhanced sampling technique using our st-CpHMD methodology (REUS-CpHMD). Six Ala-based pentapeptides (Ala_2_–X–Ala_2_) were constructed with the middle amino acid changed to one of the pH-titratable residues (Asp, Glu, Cys, Tyr, His, Lys) and with both terminals capped. Additionally, an Ala pentapeptide for each terminal was built, with either the N- or C-terminus uncapped.

### pHRE Simulation Settings

Starting conformations for all pHRE pentapeptide simulations were taken from the already published CpHMD study.^30^ These starting systems consisted of the pentapeptide in a 128-DMPC lipid bilayer, which were energy minimized in three steps. The first two steps employed steepest descent and a low-memory Broyden–Fletcher–Goldfarb–Shanno algorithm without constraints, while the final step used steepest descent with all bonds constrained using the LINCS method. The initialization of these conformations was performed in four steps of 100, 150, 200, and 250 ps, setting the v-rescale thermostat^59^ to 300 K and relaxation time of 0.1 ps on the first step, and on the second step turning on the Berendsen barostat^60^ to 1 bar in a semi-isotropic setting with a compressibility of 4.5 × 10^–5^ bar^–1^ and a relaxation time of 2.0 ps. Sequential position restraints were applied with a strength of 1000 kJ mol^–1^ nm^–2^ to all solute atoms, solute heavy atoms, and P atoms for the first, second, and third step, respectively. The final step applied position restraints only to the P atoms with a force of 10 kJ mol^–1^ nm^–2^. The pHRE simulation parameters followed those published in the CpHMD work, using a modified version of the GROMACS package version 4.0.7 ^61,62^ to include ionic strength as an external parameter.^58^ During production, long-range electrostatics were treated with the generalized reaction field method^63^ using a relative dielectric constant of 54 ^64^ and an ionic strength of 0.1 M.^58^ The simulations used a 2 fs integration step and an 8/14 Å twin-range cutoff scheme, updating the neighbor list every 5 steps. The v-rescale thermostat^59^ was used to maintain a temperature of ∼300 K. Separate couplings were applied to solute and solvent, and a relaxation time of 0.1 ps was used. The Berendsen barostat^60^ maintained the pressure at 1 bar with a semi-isotropic compressibility of 4.5 × 10^–5^ bar^–1^ and a relaxation time of 2.0 ps. The SETTLE algorithm^65^ constrained all waters, while all other bonds were constrained by the P-LINCS algorithm.^66^

A total of 5 replicates of 150 ns were produced for all amino acids at 4 pH values around their experimental p*K*_a_ in water (shown between parentheses). pH 3, 4, 5, and 6 were used for the acidic amino acids Asp (3.94), Glu (4.25), and C-ter (3.67); pH 8, 9, 10, and 11 for Cys (8.55) and Lys (10.40); pH 9, 10, 11, and 12 for Tyr (9.84); pH 4.5, 5.5, 6.5, and 7.5 for His (6.54); and pH 5, 6, 7, and 8 for N-ter (8.00).^43,44^ The narrowing of the pH range on the ionized-site side was intentional to save computational time, since these were only needed in the well-solvated-exposed regions, which are not relevant to this work. Every 20 ps simulation time (starting at 10 ps, to misalign from the protonation change attempts, which start at 0 ps), a replica exchange was attempted between two adjacent pH values, with the exchange accepted according to a Metropolis criterion.^67^

### US-CpHMD Simulation Settings

The US-CpHMD simulations used the *z*-axis of the system box as the reaction coordinate, setting the average position of the phosphorus atoms of the lipid monolayer closest to the pentapeptides as the reference (zero). Umbrella windows were defined by dividing the system into 2 Å intervals, and the range for each pentapeptide simulation spanned from 20 Å to the maximum insertion of the pentapeptide observed in the pHRE simulations (Figure 1). Since these peptides do not permeate lipid bilayers, there is no need to reach the membrane center, as in membrane permeability studies.^39^ The maximum insertion was obtained by comparing the z-position of the titratable group of each pentapeptide and the average position of the phosphorus atoms of the closest membrane leaflet in pHRE simulations (Figure S1 of the Supporting Information). Due to the different polarities and sampled spaces of each pentapeptide, distinct maximum insertion values were defined for each system.

**Figure 1:**
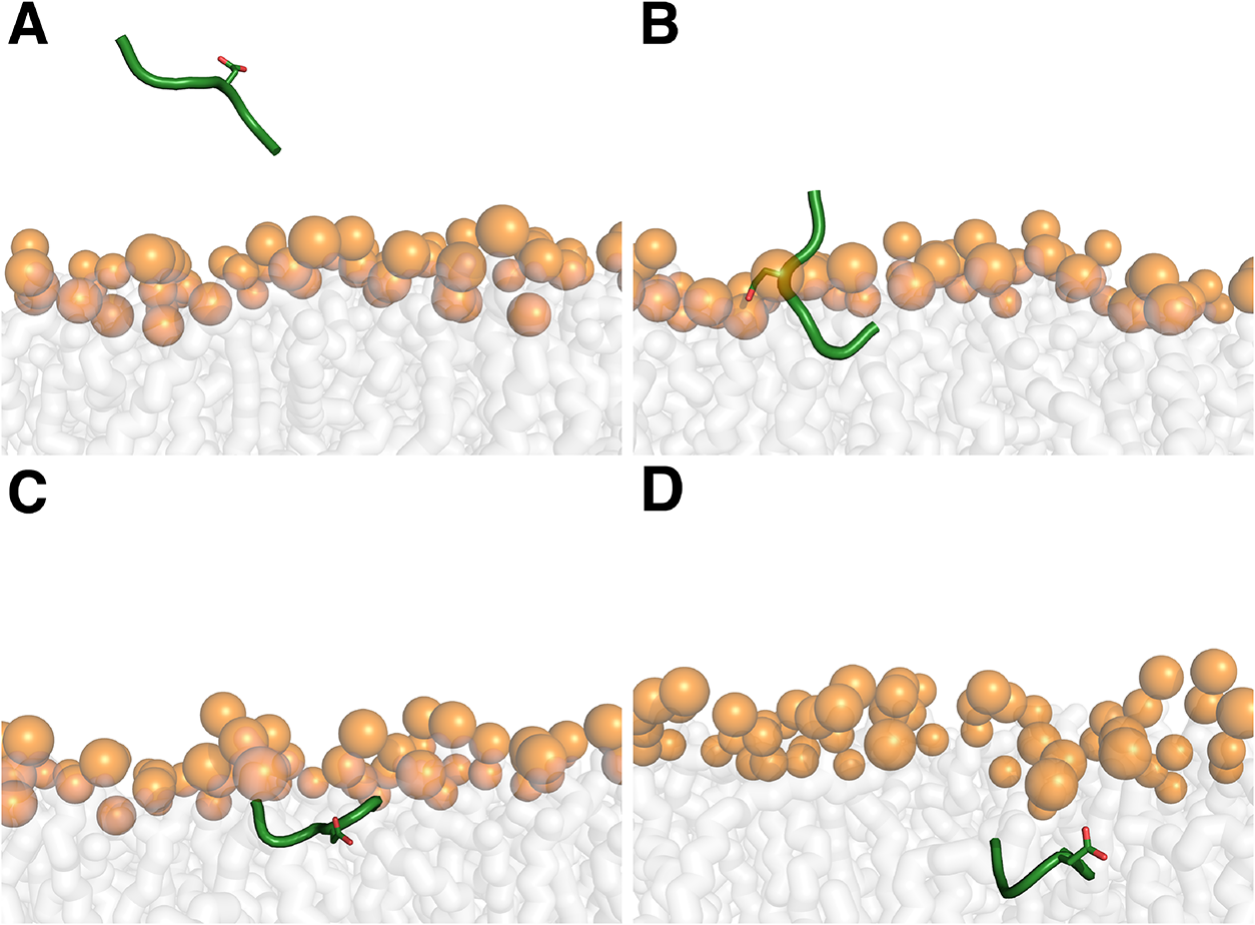
Asp-containing pentapeptide representation at membrane insertion +18 (A), 0 (B), −4 (C), and −10 (D) Å measured through the system box *z* axis using as reference the lipid phosphorus atoms. The pentapeptide is shown as a green cartoon, with the Asp residue displayed as sticks. The phosphorus atom in the lipid is depicted as an orange sphere, while large, transparent gray sticks represent the acyl chains.

The Glu, C-ter, and N-ter pentapeptides reached a maximum insertion of −12 Å, Asp, Tyr, His, and Lys pentapeptides reached −10 Å, and Cys reached only −8 Å insertion. Starting conformations for each umbrella window were obtained from the pHRE simulations, extracting conformations at each defined insertion US window. Preferentially, starting conformations have been extracted from different replicas. However, in a few cases, the most frequently inserted positions were sampled using only a single pHRE replicate, necessitating the collection of starting conformations from different times within the same replica. For the water-phase umbrellas, although pHRE simulations lacked conformational sampling beyond 20 Å, we used the furthest position and allowed the biasing potential to reach the defined insertion position. All umbrella windows had a biasing force constant of 1000 kJ mol^–1^ nm^–2^ applied to atoms of the charge group of each titratable residue of the pentapeptide: the carboxylic carbon for Asp, Glu, and C-ter, the sulfur for Cys, the oxygen for Tyr, the amine nitrogen for Lys and N-ter, and finally the center of geometry between Nϵ and Nδ atoms in His. Three replicates with 100 ns simulation time for each umbrella were produced for all amino acids. The same 4 pH values studied in pHRE were used, with two additional values (US-ext) added to improve sampling of ionized species. For anionic residues, 2 higher pH values were added, while 2 lower pH values were added for the cationic residues.

Simulations were performed with GROMACS 5.1.5 software package^68^ with GROMOS 54A7 force field.^69–71^ Temperature was kept at ∼300 K with the v-rescale thermostat^59^ and pressure was maintained by the Parrinello–Rahman barostat,^72^ at a pressure of 1 bar with a semi-isotropic compressibility of 4.5 × 10^–5^ bar^–1^ and a relaxation time of 2.0 ps. The SETTLE algorithm^65^ constrained all waters, and all other bonds were constrained by the P-LINCS algorithm.^66^ In US-CpHMD production, long-range electrostatics were treated using the particle-mesh Ewald and Verlet scheme. A single cutoff of 1.0 nm was used for both Coulombic and van der Waals interactions, in line with the method development.^55^ We attempted a compromise by maintaining the conditions described in the original papers for each approach, while also migrating to newer versions of GROMACS, which have since discontinued all group-based methods. Additionally, we have not observed major differences between the GRF and PME long-range electrostatic treatments across several systems,^73^ and both have been shown to model a fluid lipid bilayer.^74^ Nevertheless, to quantify the impact of this difference, we have performed control simulations with an anionic (Asp) and cationic (His) pentapeptide, using pHRE on 3 replicates of 150 ns, to compare the two approaches.

### REUS-CpHMD Simulation Settings

The replica-exchange simulations were performed with the same settings as in US-CpHMD. They were run only for the Asp pentapeptide system at pH 5 to test its integration and performance, and compare US-CpHMD with the REUS integration. The Asp simulations were extended to 250 ns across 3 replicates in both methods. REUS-CpHMD was run under the same umbrella sampling scheme as regular US, with windows ranging from +20 to −12 Å and 2 Å intervals. In this method, two different biasing forces were applied, from 20 to 8 Å a lower biasing force (500 kJ mol^–1^ nm^–2^) was applied since in these windows there no strong interactions with the lipid bilayer, from 6 to −12 Å a stronger (1000 kJ mol^–1^ nm^–2^) biasing force is applied, since in this range some regions are hard to sample. The introduction of two different biasing potentials enabled the evaluation of the impact of a smaller biasing force on the exchange acceptance ratio. This was applied only in the water-phase region, where sampling is more homogeneous, and all methods converged with no significant impact on the PMF profile or sampling performance. Additionally, another REUS scheme with a 1 Å window separation was produced to increase the number of exchanges between umbrellas. However, due to limitations in the number of computer cores available in the simulations, our umbrella scheme spanned only −10 to 12 Å.

In REUS, exchanges are attempted each 20 ps between two adjacent umbrella windows at the same temperature and pH values, *W*_*m*_(*i*) and *W*_*n*_(*j*), accepting the exchange using the Metropolis criterion between the harmonic biasing potential *V* of each umbrella window (eq 1)­

1 $$V_{m}\left(i\right)=\frac{k_{m}}{2}{\left(\xi_{i}-\xi_{m}\right)}^{2}$$

with ξ_*i*_ as the reaction coordinate value of the frame and ξ*_m_* as the reference reaction coordinate value of *W*_*m*_. *k*_*m*_ is the force constant applied at the umbrella window. Following a Metropolis criterion, the exchange probability (ω) is described by the following equation

2 $$\omega \left(W_{m}\to W_{n}\right)=\{\begin{array}{l} 1\;\mathrm{for}\;\Delta \leq 0,\\ e^{-\Delta }\;\mathrm{for}\;\Delta >0, \end{array},w\mathrm{ith}\;\Delta =\beta \left(V_{m}\left(j\right)-V_{m}\left(i\right)-V_{n}\left(j\right)+V_{n}\left(i\right)\right)$$

In this formalism, we can frequently exchange conformations between adjacent umbrellas in their overlapping regions, thereby significantly improving overall sampling of the process.

### PB/MC Settings

The PB calculations for all methods used DelPhi Version 5.1,^75^ with partial charges taken from ref 76 and radii derived from the Lennard-Jones parameters of the GROMOS 54A7 force field.^69,70^ The ionic strength and ion exclusion layer were set to 0.1 M and 2.0 Å, respectively. A probe of radius 1.4 Å was used to define the molecular surface. To ensure continuity in the membrane surface, 5% of the box vector dimension was added in the *x* and *y* direction.^71^ The dielectric constants of 80 and 2 were used for water and solute (peptide and membrane), respectively. Periodic boundary conditions were applied in both the *x* and *y* directions for the potential calculation on a coarser grid, with relaxation parameters of 0.2 and 0.75 for the linear and nonlinear PB equations, respectively. The background contributions and pairwise interaction^71^ calculations used a cutoff of 25 Å. The convergence threshold value was set to 0.01 *k*_B_ *T*/*e*, and a cubic grid of 81 grid points was used. The calculations were performed with a two-step focusing method:^77^ the coarser grid spacing was ∼1 Å, which is four times the size of the focus grid, ∼0.25 Å. The PETIT program v.1.6 ^78^ was used to perform 10^5^ MC steps at 300 K to sample protonation states.

### Simulation Analysis

In line with our previous paper,^30^ we discarded the first 25 ns as the equilibration time and analyzed the equilibration properties from that point onward. The PMFs for both the US and REUS-CpHMD simulations were calculated with the WHAM tool in the GROMACS package^79^ from equilibrated data across all 3 replicates per pH. The error values for the PMF profiles were calculated using the jackknife leave-one-out approach, which involved combining PMF pairs across the three replicates for each pH value within each pentapeptide. This error provides a more accurate description of the variability between replicas, which is not captured by the WHAM fit errors.

To accurately capture the insertion of the pentapeptide, it is crucial to consider membrane deformations. These calculations were performed using the MembIT tool.^80^ In these calculations, only the lipid phosphate groups (including the phosphorus and its 4 bound oxygen atoms) within a 6 Å radius of the titratable site of the pentapeptide were considered in the insertion calculation, provided that at least 5 atoms were present. The reference atoms for the titratable amino acid sites are the carboxylic carbon for Asp, Glu, and C-ter, the sulfur for Cys, the oxygen for Tyr, the amine nitrogen for Lys and N-ter, and finally the center of geometry between Nϵ and Nδ atoms in His.

Membrane deformation was also calculated using the MembIT tool, using the same reference for the titratable sites. For local membrane deformation, the tool compares the average positions of lipid phosphate groups within 6 Å of the titratable site with those of bulk lipid phosphates more than 15 Å away, values that were optimized in previous work for similar systems.^31,32^ These local deformations were calculated for each pH value and umbrella window, and data from the 3 replicates were used to compute the standard error of the mean.

### p*K*_a_ Profile Calculations

In membrane systems, the protonation behavior of titratable groups is not well captured by a single p*K*_a_ value because of interactions with distinct lipid regions. To decompose this membrane effect on the titrating groups, we have characterized the p*K*_a_ along the membrane normal. At each simulation time step, it is crucial to determine the correct insertion and protonation states of our titratable residue. The protonation state is directly obtained from the simulations and is established in each PB/MC calculation. The insertion of our titratable residue was determined using the MembIt tool, which identifies lipid phosphate groups within a 6 Å radius of the titratable site and incorporates at least 5 atoms in the reference. Combining data from all replicates and pH values, we have sliced the *z*-axis of our system and calculated the p*K*_a_ values of the pentapeptides and the population of ionized species as a function of membrane insertion. The latter were obtained by slicing the insertion range, generated by the MembIt tool, into 0.2 Å bins from −15 to 25 Å and counting the number of ionized configurations sampled across all replicates and pH values for each pentapeptide system. The p*K*_a_ profiles were obtained by scanning the insertion range from −12 to 22 Å with a 1 Å bin size and a sliding step of 0.25 Å. At each step, the frames within the bin length of insertion are gathered from the 3 replicates, and all pH values are considered. The corresponding protonation states of the site are then averaged per pH and fitted to a Henderson–Hasselbalch equation to estimate the p*K*_a_ value at each insertion bin. Several criteria must be met for the fitting to occur: each protonation state must have at least 50 points, obtained from at least two pH values and two different replicas. Additionally, curve monotonicity must be respected. The same protocol was applied to all three methodologies, CpHMD, pHRE, and US-CpHMD, to obtain each pentapeptide p*K*_a_ profile. Error bars for each calculated p*K*_a_ value were obtained using the Bayesian bootstrap with 1000 bootstraps from our average protonation samples to mitigate fitting issues.^33^

## Results and Discussion

### Sampling Comparison between CpHMD vs pHRE vs US-CpHMD

We have simulated and analyzed the proton-binding affinities (p*K*_a_ values) of all pH-sensitive amino acid residues when inserting into a DMPC membrane. This relatively simple system is ideal for testing the ability of our various computational methodologies to capture the complex protonation and conformational space of this process. Since p*K*_a_ values are highly sensitive to the environment surrounding the titrating molecule, it is crucial to correctly capture and describe the insertion of our titrating group into the DMPC membrane. Possible membrane deformations, caused by the approximation or insertion of the pentapeptides, need to be accounted for. Only the nearby lipids surrounding the inserting molecule should be considered in the insertion calculation. In this work, we utilized the MembIT tool, developed within our group,^80^ to perform the insertion calculation, focusing solely on the titratable group of the pentapeptide.

Sampling quality is always a crucial issue when performing MD simulations of biological processes. In the pentapeptide p*K*_a_ profiling along membrane insertion, two distinct sampling problems arise. First, sufficient configurations are required at all insertion positions, which can be obtained via either long simulations or enhanced sampling methods (e.g., umbrella sampling). Second, we have protonation sampling restrictions, especially in environments that overstabilize one of the species. To overcome the protonation sampling problem, a pH Replica Exchange (pHRE) methodology was introduced within the CpHMD framework.^50^ To address these limitations separately and compare methodologies, we employed three methods in this work: the stochastic titration CpHMD (CpHMD), which serves as our reference methodology; pHRE, which introduces pH replica-exchanges; and US-CpHMD, which uses biasing potentials to enhance sampling along the membrane insertion axis.

To evaluate the performance of each method, we calculated the probability distributions of our pentapeptide membrane insertions (Figure S1 in the Supporting Information). Unsurprisingly, the US-CpHMD methodology sampled the insertion space of our pentapeptides more effectively. For the CpHMD and pHRE methods, the number of insertion regions sampled per residue is highly pH-dependent. For cationic residues, we observed improved sampling of more-inserted regions, especially at higher pH values, where the neutral form is prevalent. Nevertheless, there are exceptions, especially in the CpHMD simulations (see Lys at lower pH values), where the charged species can become kinetically trapped, significantly affecting sampling quality across pH values. In the anionic residues, the same pH behavior was observed, but in the opposite pH range, with the inserted positions being more populated at lower pH values. This reinforces the overall idea that pH-sensitive molecules tend to neutralize as they move toward more apolar media. This alteration in the neutral and ionized populations directly shifts the p*K*_a_ of the molecule, which must be accounted for when analyzing these biological processes.

To obtain p*K*_a_ values of each amino acid along the insertion axis, we should consider that better conformational sampling within the insertion range may not correlate with more robust estimations of the p*K*_a_values. Indeed, the limiting factor to compute these values is the abundance of the ionized state along the insertion axis (Figure 2). Among the explored methods, we observe improvements in sampling ionized states deep in the membrane, with CpHMD∼ pHRE < US < US-ext, although some of the differences are methodological by construction. When comparing only the regular 4 pH values, we see that the improvement in pHRE over CpHMD is marginal, and some gain is achieved with US-CpHMD, primarily in some of the anionic residues (Cys, Tyr, and C-ter). The smaller contribution of enhanced sampling methods to sampling cationic residues may be due to the slightly negative character of the phosphate/glycerol region in phospholipids, which tends to favor sampling of the pentapeptide’s ionized species. When deconvoluting the ionized populations by pH, it becomes clear that the pH values that promote ionization make the major contribution to these profiles (Figure S2 of the Supporting Information). As expected, the two additional pH values (US-ext) significantly improve the sampling of ionized species in most pentapeptides. These results show that the choice of pH range can also be crucial when establishing such a simulation protocol. Additionally, when discussing sampling in biased simulations, we should evaluate the effect of the biasing potential by reweighting the populations explored (Figure S3 in the Supporting Information). Our results show that the tight binning procedure adopted in the non-reweighted data and the overall homogeneity of our sampling lead to an almost negligible effect of the reweighting procedure on the ionized population distributions. Since we present our p*K*_a_ and membrane deformation data as insertion profiles, we leveraged this similarity and presented the data without reweighting.

**Figure 2:**
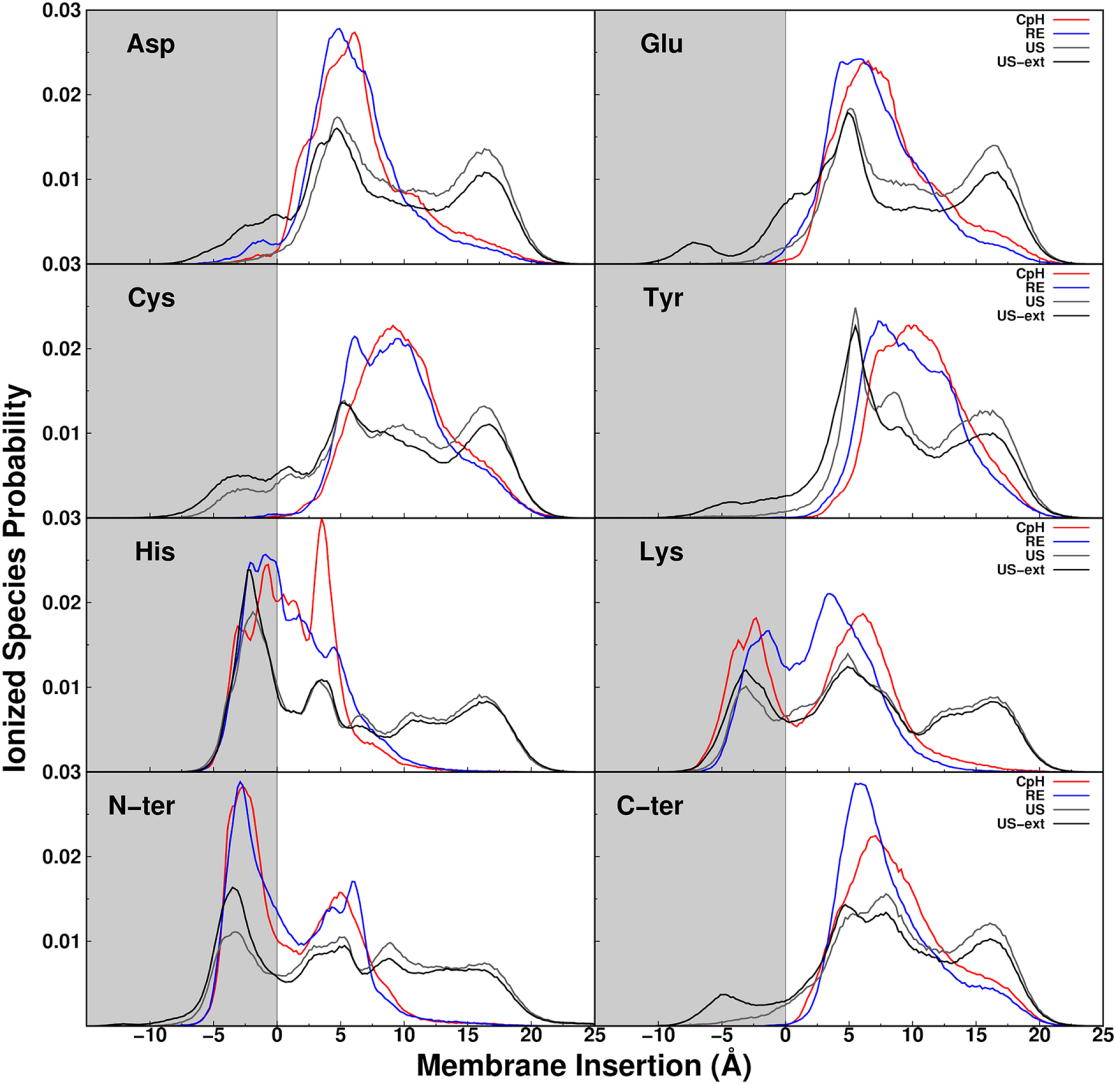
Probability of the ionized species along the membrane insertion axis measured through MembIT, for all titratable groups of the 8 pentapeptide systems, Asp, Glu, Cys, Tyr, Lys, His, N-ter, and C-ter. Populations were calculated using all pH values and the three replicates, employing the distinct methods: CpHMD (red), pHRE (blue), US-CpHMD (gray–same pH values, US; black–with two extra pH values, US-ext). The region inside the membrane is shown as a gray area in the graph.

Despite the sampling improvement of US-CpHMD, applying a biasing potential to the most frequently inserted positions can also lead to high-energy conformations that require careful analysis. One such example is the ionized population observed in Glu at −8 Å insertion. These pentapeptide/membrane conformations are usually the result of membrane deformations where lipids adopt unusual configurations to stabilize the pentapeptide’s charge. In a curious case, we observed a choline group inverted to stabilize the negative charge of glutamate in deeper membrane regions (Figure S4 of the Supporting Information).

There is also a difference in the long-range electrostatic treatment between GRF (CpHMD and pHRE) and PME (US-CpHMD), which may affect the ionized population distributions. We tested this possibility using Asp and His in pHRE simulations and noticed that in the Asp pentapeptide, the PME distribution is slightly reduced in deeper membrane insertion regions (Figure S5 of Supporting Information). This behavior may also arise from differences in membrane fluidity between the two long-range electrostatic treatments (Figure S6 of Supporting Information). Overall, this effect slightly influences the p*K*_a_ profiles (Figure S7 in the Supporting Information), therefore, the observed sampling differences should not be attributed solely to the enhanced sampling technique employed. Nevertheless, our method comparison indicates that, although it entails a higher computational cost, US-CpHMD yields improved sampling of ionized populations in deeply inserted membrane regions.

### Pentapeptide p*K*_a_ Variation with Membrane Insertion

To study the p*K*_a_ shifts induced by the pentapeptide membrane insertion, we have obtained the p*K*_a_ profiles of all titratable amino acids with all three methods studied (Figure S8 of Supporting Information). The CpHMD p*K*_a_ profiles were previously published^30^ using a less robust membrane insertion protocol based on the closest lipid phosphorus atom, as a reference. This provides a crude estimate of membrane insertion, and we have recalculated it using the MembIT tool, including all lipid phosphate atoms (phosphorus and the four oxygen atoms bound) within a 6 Å radius of the titratable residue, thereby providing a more robust insertion measurement.

The three explored methods show a clear ability to obtain p*K*_a_ predictions upon insertion, further cementing the relation CpHMD < pHRE < US in sampling capability. In this approach, we used the US-ext data, which demonstrated a clear improvement in the attainment of ionized populations. Curiously, these p*K*_a_ predictions indicate that CpHMD and pHRE have diverged in their sampling capabilities, highlighting limitations in CpHMD configurations that prevent greater accuracy. This may be due to insufficient ionized species across multiple pH values, across different replicas, and to adherence to a monotonicity criterion for protonation. Overall, improved sampling in US-CpHMD often yields a more comprehensive p*K*_a_ profile than other methods. For some anionic amino acids, the improvement is significant, and the profiles are calculated more deeply within the membrane. However, for the cationic residues, we observe similar results using US and pHRE. All methods capture very similar p*K*_a_ shifts along membrane insertion, differing only in their ability to sample ionized conformations at deeper insertions.

Using the US-CpHMD data, we can calculate the p*K*_a_ profiles of all amino acids (Figure 3). We observe that the membrane begins to exert an effect on the amino acids p*K*_a_ values at a distance of ∼8 Å, thereby stabilizing their neutral states. For anionic residues, this shift increases the p*K*_a_ values, while for cationic amino acids, a decrease is observed. This trend continues until we cannot capture sufficient ionized states to calculate the p*K*_a_ of the amino acids. At ∼−6 Å, the titratable groups reach the glycerol ester region (the beginning of the lipid acyl chains), making estimation of p*K*_a_ values much more difficult due to the environment’s apolar nature. In this region, water becomes scarce, and the titratable groups lose their ability to exchange protons; in other words, the concept of macroscopic pH breaks down. In principle, we could continue to estimate proton binding affinities beyond this insertion point, as the method allows, but they would be of limited usefulness. The p*K*_a_ profiles obtained here are in excellent agreement with those published 10 years ago,^30^ although our sampling is significantly more extensive. This improvement is noticeable in both the size of the error bars and the membrane depth of the profiles.

**Figure 3:**
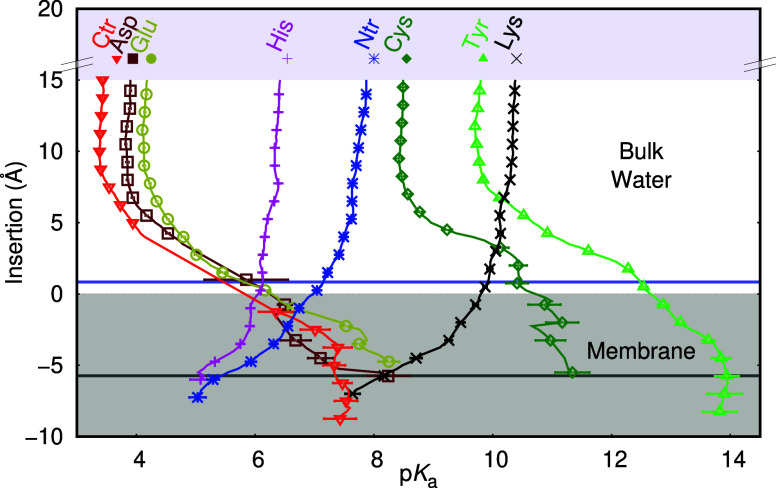
p*K*_a_ values along the membrane insertion coordinate calculated with US-CpHMD for all amino acid residues and the two termini. Please note that this membrane insertion accounts for the first interacting layer of phosphate atoms, rather than the entire monolayer (see Methods section for details). Aqueous p*K*_a_ values are shown on the top for reference. The gray area represents the membrane region. A horizontal blue line indicates the average position of the choline nitrogen atoms at approximately 1 Å, and a dark gray horizontal line marks the start of the acyl chain region. Error bars were computed using the bootstrap method with 1000 bootstraps.

The p*K*_a_ shifts obtained for titratable amino acids incorporated in pentapeptides can be easily extrapolated for other groups, including membrane-permeating drugs,^39,81^ titrating lipids,^37,71,82,83^ membrane-inserted peptides,^27,31–33,84^ and membrane proteins.^55,85^ This result underscores the importance of studying pH in the context of permeant molecules, as charge loss is crucial for accurately describing water uptake into a lipid environment.

The insertion of molecules into the membrane naturally induces bilayer deformations, thereby facilitating their adsorption and insertion. Although the MembIT insertion calculation accounts for the membrane’s natural deformation, it remains important to evaluate the local membrane deformation induced by peptide insertion to validate the health of our system. Local deformation was calculated with MembIT by comparing the position of the phosphate group of phospholipids within a 6 Å radius of the titratable molecule and the position of far away (bulk) phospholipids (Figure 4). As the pentapeptide approaches the membrane, at around 8 to 10 Å, we observe a positive membrane deformation, as the lipid/pentapeptide attraction results in a slight protrusion of the lipids, since we are restraining the pentapeptide in the *z* coordinate. This effect is consistent with the observed region where the p*K*_a_ begins to shift, highlighting the mutual influence between the membrane and the pentapeptide. With membrane insertion, we observe negative deformation values, indicating that lipids shift downward upon adsorption of our pentapeptides. There is a tendency toward larger deformations around cationic residues, which may be due to greater difficulty in desolvating them. Another important trend is a slight increase in negative membrane deformation at pH values far from the pentapeptide p*K*_a_, when the charged state is favored. This indicates that pH modulates the permeability of titratable molecules and affects membrane integrity by balancing the populations of their ionized forms.

**Figure 4:**
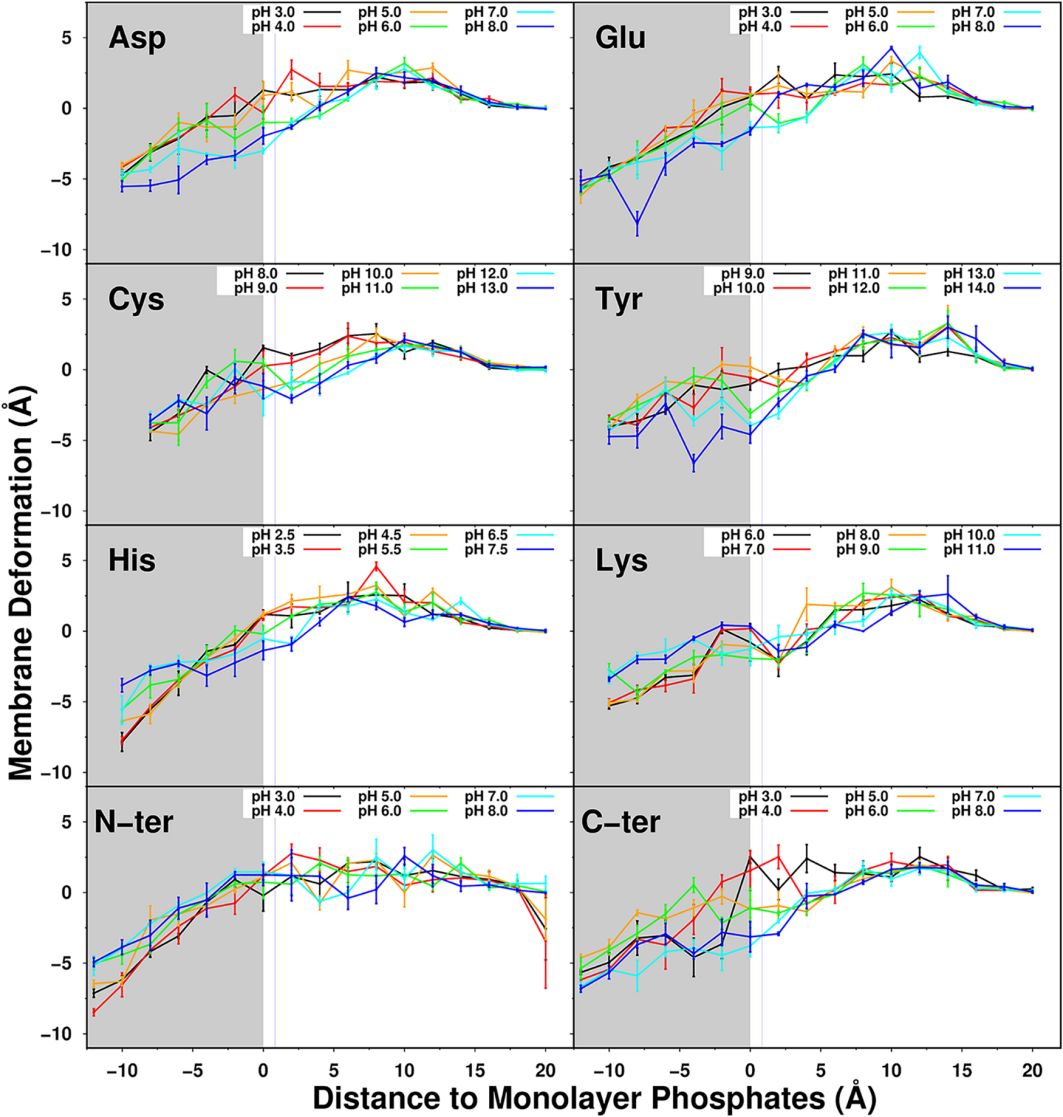
Local membrane deformation induced by pentapeptide insertion at different pH values. Calculation performed by MembIT using the average position of lipid phosphates in 6 Å radius around the pentapeptide compared to the (bulk; >15 Å) lipid phosphates. Values presented are obtained from averaging the membrane local deformation at each umbrella window, and error bars are calculated with the standard error of the mean. The gray area represents the membrane region. A vertical blue line indicates the average position of the choline nitrogen atoms at approximately 1 Å, and the dark gray line marks the start of the acyl chain region.

From the US-CpHMD data, we can calculate PMF profiles at different pH values for all titratable systems (Figure 5). These profiles were calculated using the reaction coordinate of the US rather than the reference used in our membrane insertion protocol (the distance along the *z*-axis to the average of the closest monolayer phosphate groups). Naturally, this approach does not account for local membrane deformations, and comparisons with p*K*_a_ profiles should be made with caution. The PMFs show that pentapeptides in water are attracted to the membrane until they contact it. From that point, we observe an energy barrier that begins at different membrane insertions and varies in magnitude with the degree of ionization of the titratable group. In some cases, the PMF profiles do not follow a perfect trend with pH, but these deviations are within the method’s error bars (Figure S9 of the Supporting Information). At pH values where the amino acids predominantly exist in a neutral state, we observe a steady negative slope in the PMF up to more inserted regions. When pH favors the charged state, we observe an increase in the energy barrier to crossing the membrane and a shift in the preferred interaction regions between oppositely charged species. Specifically, choline groups interact with anionic sites, and phosphate groups interact with cationic ones.

**Figure 5:**
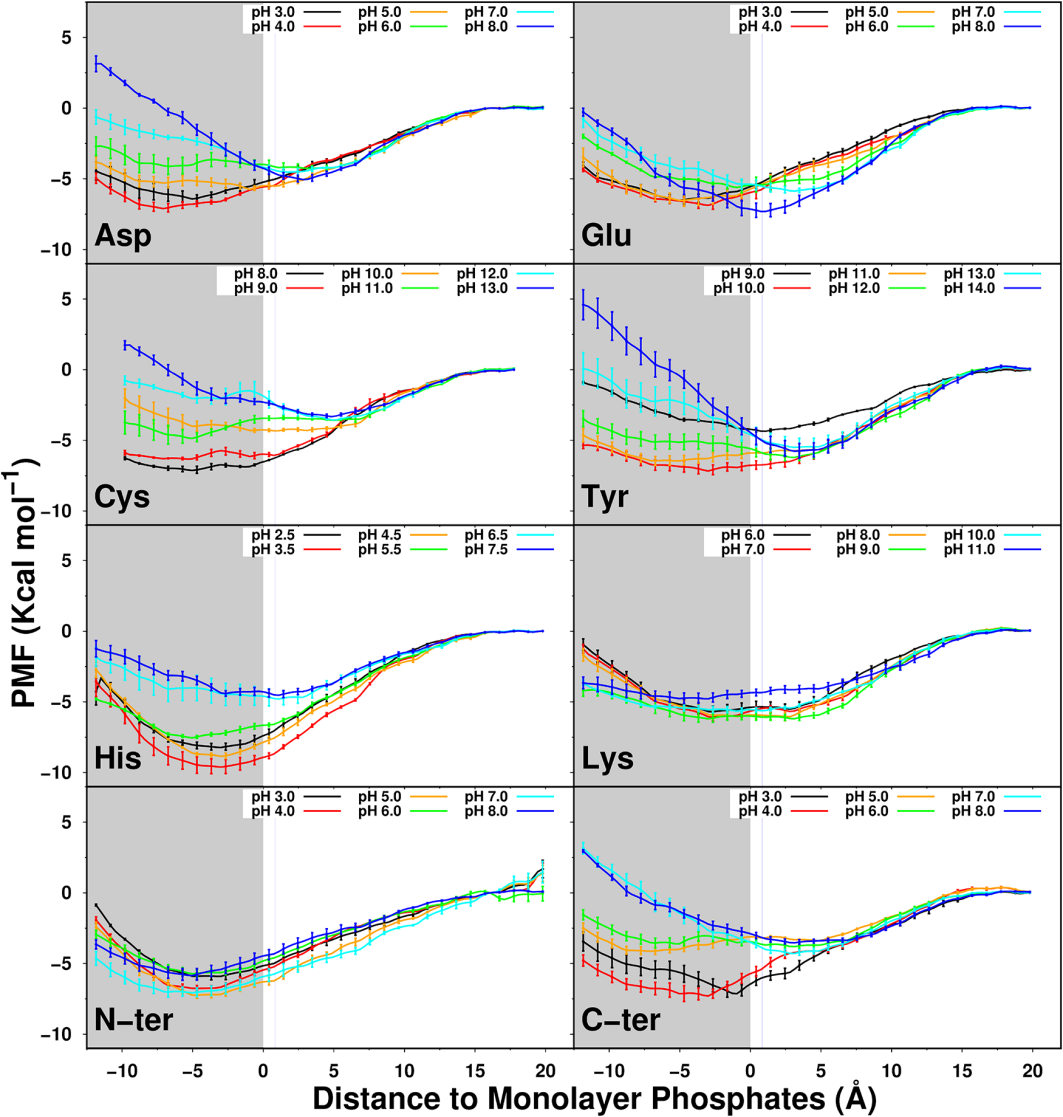
pH-dependent PMF profiles obtained from US-CpHMD simulations for all pentapeptides. The gray area represents the membrane region. A vertical blue line indicates the average position of the choline nitrogen atoms at approximately 1 Å, and the dark gray line marks the start of the acyl chain region. Error bars are shown every 3 points, calculated with the jackknife leave-one-out approach on all replicates (Figure S9 of Supporting Information).

The PMF comparisons between residues are always complex since these are affected by the % of ionization and proton affinity of the groups, which is different depending on the pH value. A comparison is only possible when considering the protonation profiles resulting from the insertion of each species. For the case of Lys versus His, although both are positively charged at pH < p*K*_a_, the populations of their charged states as they approach the membrane differ, because their p*K*_a_ values are very different and shift differently when interacting with the membrane. The distinct characteristics of the two residues (an imidazole in His and an amine in Lys) naturally lead to different polar properties, desolvation profiles, and membrane interactions, all of which affect the resulting PMF profile. For this reason, a direct comparison of PMF/pH values between residues is not straightforward. Nevertheless, an interesting case is the comparison between the PMF profiles of Asp and Glu pentapeptides at pH 8.0 (Figure 5). We would expect Asp to have a slightly higher energetic barrier to membrane entry due to its lower p*K*_a_. However, the larger difference hints at another factor: an out-of-trend membrane deformation in one of the Glu deeply inserted umbrella windows at pH 8.0 (Figure 4), which might have artificially lowered the energy barrier estimated by the WHAM method. This highlights the high sensitivity of these PMF calculations to local membrane deformations.

### Coupling a Replica-Exchange Scheme to US-CpHMD

Here, we also propose an extension of the US-CpHMD method, based on an umbrella-window replica-exchange scheme (REUS-CpHMD). In this new st-CpHMD implementation, the umbrellas with the biasing potential closest to the adjacent umbrella window can exchange their reference positions, increasing sampling of the histogram overlap regions. To directly compare the US and REUS methodologies, we have applied both methods to the Asp pentapeptide system, extending the pH 5 simulations to 250 ns across 3 replicates.

Considering that REUS improves mostly the sampling in the region between the US windows, it comes as no surprise that the ionized populations between US and REUS are quite similar (Figure 6A). The two-peak distribution observed results from a depression in peptide abundance at ∼10 Å, triggered by the strong affinity between the peptide and lipids, which pulls them together once they enter the nonbonded interaction cutoff (10 Å). Nevertheless, the use of the replica exchange scheme yields a smoother population distribution and improved sampling between US windows, which also positively impacts the robustness of protonation along the membrane insertion (Figure 6B).

**Figure 6:**
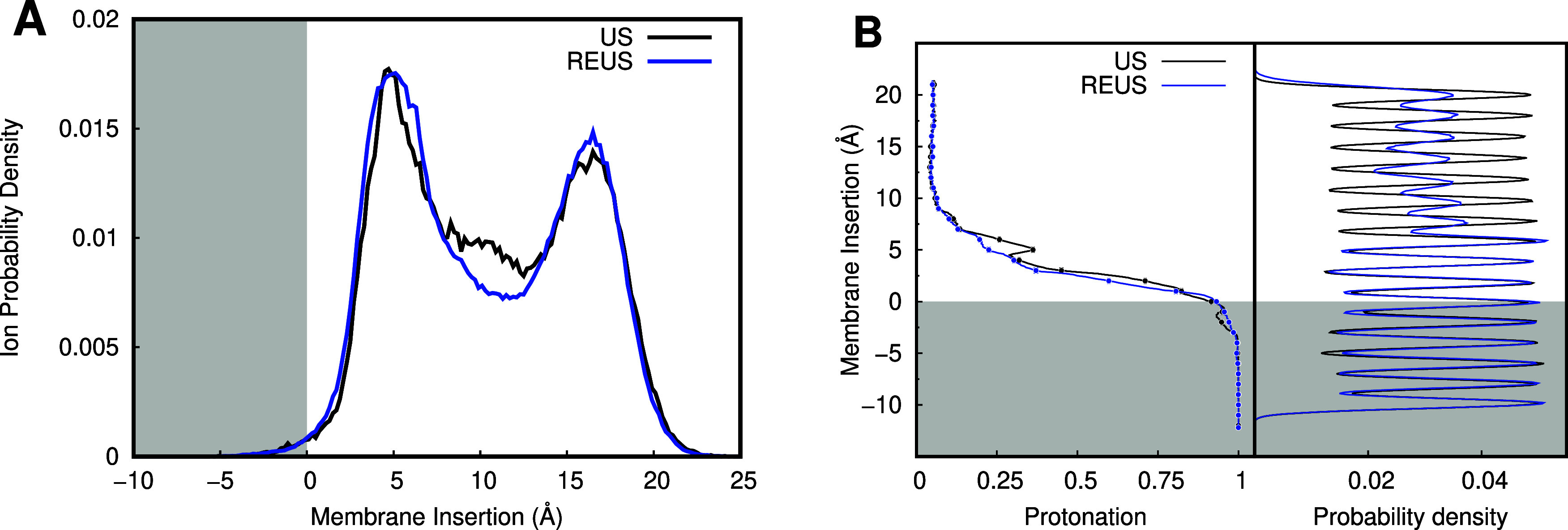
Methodology comparison between US-CpHMD (black) and REUS-CpHMD (blue) through ionized populations (A) and protonation profiles along membrane insertion (B). Data are shown for the Asp-containing pentapeptide at pH 5.0. Protonation profiles calculated with the reaction coordinate sliced with 0.01 nm bins. The total conformation histogram for both methods is shown on the right. The gray-shaded areas correspond to the membrane-inserted regions.

In REUS simulations, the number of accepted exchanges is a key metric for assessing the success of the sampling improvement. We counted the number of exchanges between umbrellas and, unsurprisingly, observed an increase in exchanges with smaller biasing potential (from 8 Å upward) (Figure S10 of the Supporting Information). Such a result highlights the impact of reducing the biasing force on the acceptance ratio. However, this optimization must be approached carefully to avoid conformational entrapment in energy minima, which would severely penalize US sampling in high-energy regions. The changes in the pentapeptide’s degrees of freedom as it inserts into the membrane (region with 1000 biasing force) do not appear to significantly affect the exchange rate, as they are constrained by both the conformational space available to sample and by their modulation with the bias force strength. To increase the number of exchanges in our simulations, we have developed a new REUS scheme in which umbrella windows are separated by only 1 Å. The smaller separation between umbrellas in the new scheme boosts exchanges by a factor of 10 to 25, accompanied by improved sampling, but at the cost of requiring more umbrella windows.

To evaluate the impact of REUS on final PMF convergence, we calculated PMF profiles with blocks of 150 at 20 ns time steps, starting from 0 ns and extending to 100 ns (Figure S11 of Supporting Information). In all three methods, US, REUS-2 Å, and REUS-1 Å, we observe that a time of around 20 to 40 ns is sufficient to achieve convergence of the PMF profile. However, in peptide/membrane systems, lipids often require hundreds of nanoseconds to equilibrate their positions. Therefore, although we observe an apparent convergence, this may still be affected by configurational heterogeneity between the pentapeptide and the surrounding lipids. This is particularly evident in the US simulations, where there were no exchanges between umbrellas to mitigate those heterogeneities.

Comparing the converged PMF profiles (100–250 ns segments), we observe that REUS with a 1 Å separation can output a smooth PMF profile, describing the energy profile of inserting an aspartic pentapeptide into a membrane with high confidence (Figure 7). In the 2 Å REUS window scheme, exchanges are scarcer, leading to less efficient sampling between windows and increasing roughness in the PMF profile. In the limit of no umbrella exchanges (US-CpHMD), we observe larger deviations in the membrane-inserted regions, where sampling is less frequent. This becomes clearer when considering the jackknife errors computed for the enhanced sampling simulations. When using a simple US-CpHMD scheme, we observed PMF errors of approximately 0.4 kcal/mol for the most deeply inserted US windows. On the other hand, when applying a double-enhanced sampling REUS methodology, the errors between replicates are nearly halved by mixing conformations from different umbrellas, thereby improving the homogeneity of the sampled space.

**Figure 7:**
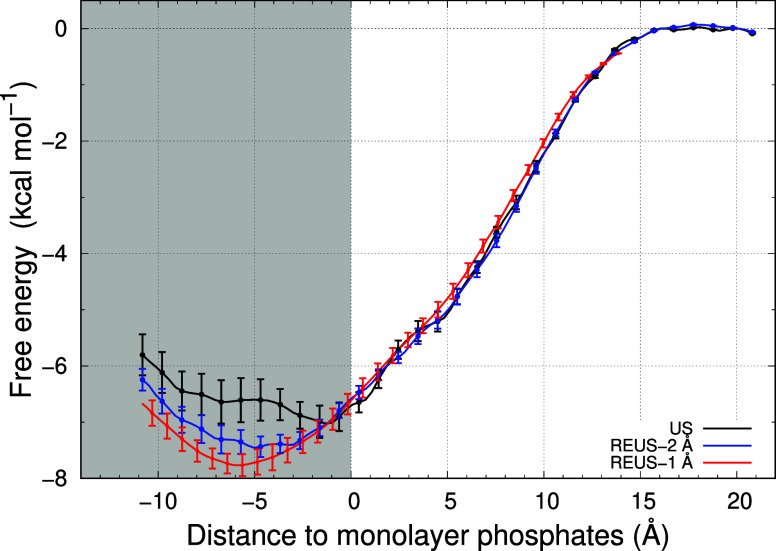
PMF profiles of US-CpHMD (black), REUS with US window separation of 2 Å (blue), and REUS with US window separation at 1 Å (red) using the last equilibrated segment (150 ns). REUS-1 Å lacks points in the 14 to 20 ns range; hence, we aligned its PMF in the water region to the REUS-2 Å data. The gray-shaded area corresponds to the membrane-inserted region. Error bars were calculated with the leave-one-out jackknife approach on all replicates and are less than 0.5 kcal/mol even deep inside the membrane (Figure S12 of Supporting Information). For the US, the errors reach a maximum of 0.4 kcal/mol, while REUS reaches errors of 0.2 kcal/mol at the most inserted positions.

In conclusion, incorporating replica exchange into US-CpHMD enhances sampling, yielding a smoother protonation profile and a PMF with lower jackknife errors across replicas. This improvement aligns with the methodology’s formalism, yielding improved sampling of regions between US windows and the homogenization of all umbrellas. The latter is significant because, with the REUS scheme, we can include contributions from every configuration in the initial setup within each umbrella. This would be equivalent to a US-CpHMD setup with 20 × 3 replicates. Notwithstanding, when examining the ionized population, both methods sample the same regions, albeit with different levels of detail. Consequently, all potential improvements to the p*K*_a_ estimations from the REUS scheme should not be due to an increase in the sampling of the ionized populations, but rather a more balanced sampling of the correct configurations in deeper regions of the membrane. This increase in sampling comes with a higher computational cost, as REUS requires more cores per node than the regular US-CpHMD scheme, primarily because it must run all umbrella windows in parallel. Such a framework is only suitable for HPC clusters with high-speed internode connections or for machines with a large number of internal processing units. Often, the additional gains from a REUS scheme can be outweighed by the high computational cost, particularly when using small- to medium-scale computational infrastructure. Fortunately, for many systems, the US-CpHMD protocol alone will suffice.

## Conclusions

In this study, we have compared the performance of enhanced-sampling CpHMD techniques in describing an alanine pentapeptide model system inserting from the water phase into a DMPC membrane. We have evaluated the p*K*_a_ profiles for six titratable amino acids, Asp, Glu, Cys, Tyr, His, and Lys, and two termini, N and C-termini. We have employed RE and US, coupled with CpHMD, to improve the p*K*_a_ profiles obtained in our previously published work.^30^ We have also used the MembIT tool^80^ in our protocol to calculate more accurate membrane insertion values for these molecules. We moved from the closest-lipid approach to determine molecular insertion to using phosphate-group atoms within ∼6 Å of our pentapeptides, thereby improving the method’s robustness. Overall, the p*K*_a_ profiles obtained became more consistent as method complexity increased, from CpHMD to pHRE to US-CpHMD.

The umbrella sampling scheme showed a clear improvement in describing the p*K*_a_ values in deeper membrane regions for all pentapeptides studied, with particular success for the anionic molecules. Equally important is the choice of pH values that promote the ionization of these sites in deeper membrane regions. Our results are also in line with the published p*K*_a_ shifts expected in the membrane environment, favoring the neutral states of the amino acids. Anionic molecules tend to shift their p*K*_a_ upward when inserted into a membrane environment, while cationic molecules shift in the opposite direction. A direct relationship between the ionization state of the titratable group and the PMF barrier to membrane insertion was also observed, highlighting the modulation of pH, ionization, and membrane partitioning of our molecules. To achieve even better sampling, a RE scheme was successfully coupled with US-CpHMD (REUS-CpHMD), enabling conformational exchanges between adjacent umbrellas. This implementation further improved the reliability and robustness of the method, both in describing protonation and energy barriers (PMF), albeit only for one system at a single pH value due to the prohibitively high computational cost of the methodology.

## Supplementary Material





## Data Availability

The GROMACS package is freely available software for MD simulations and can be downloaded at https://manual.gromacs.org/5.1.5/download.html. MembIT is an in-house-developed tool available for free at https://github.com/mms-fcul/MembIT. PyMOL v3.1 is also free software for molecular visualization and high-quality image generation. It can be downloaded from https://pymol.org. The CpHMD code is also provided inside the Supporting Information zip file.

## Notes

A methodological comparison of the computational molecular dynamics techniques explored in this work; the insertion probability density histograms for all systems at all pH values studied; a high-energy conformation of the Glu-containing pentapeptide, where distorted lipid choline groups stabilize the ionized species; p*K*_a_ profiles comparison between the different CpHMD methods for the 8 pentapeptide systems; error values computed for the US-CpHMD pH-dependent PMF profiles for all amino acids and pH values; umbrella windows exchange along membrane insertion for the two REUS-CpHMD protocols; PMF convergence over time for US and REUS protocols, and PMF error values comparison between the REUS and US-CpHMD methods (PDF)
Topologies, system configurations, parameter files, and the CpHMD code (ZIP)
