# Supplementary material for: Improving the Estimation of pK a Values for All Titratable Amino Acids at the Water/Membrane Interface
Source: J Phys Chem B. 2026 Apr 13;130(16):4270–82. doi: 10.1021/acs.jpcb.5c07807 (PMC13112345; doi:10.1021/acs.jpcb.5c07807)
Supplement: Supplementary file 1 [file jp5c07807_si_001.pdf]

Supporting Information: Improving the Estimation of pK_a Values for All Titrable Amino Acids at the Water/Membrane Interface

Nuno F. B. Oliveira,[†] Pedro B. P. S. Reis,[‡] and Miguel Machuqueiro[*,†]

†*BioISI - Instituto de Biossistemas e Ciências Integrativas, Faculdade de Ciências, Universidade de Lisboa, 1749-016, Lisboa, Portugal*

‡*Machine Learning Research, Bayer AG, Müllerstraße 178, 13353, Berlin, Germany*

E-mail: machuque@ciencias.ulisboa.pt

Phone: +351-21-7500112

Table S1: Methodological comparison between the computational molecular dynamics techniques explored in this work. Details regarding their use, sampling, and computational cost are provided for st-CpHMD, US-CpHMD, and REUS-CpHMD, with regular MD as the base reference.

Method	**MD**	**st-CpHMD**	**pHRE**	**US-CpHMD**	**REUS-CpHMD**
**Sampled Property**	Conformation	Conformation + Protonation	Conformation + Protonation	Conformation + CV + Protonation	Conformation + CV + Protonation
**Enhanced Sampling Type**	–	–	Replica exchange	Umbrella Sampling	Umbrella Sampling + Replica Exchange
**Enhanced Sampled Property**	–	–	pH replica exchanged	CV (Membrane Insertion)	CV (Membrane Insertion) + Configuration Replica Exchanged
**MD scheme**	Independent simulations	Independent simulations	pH range simulations *in tandem*	Independent simulations along CV	Multiple CV simulations *in tandem*
**Computational Cost**	0	+	++	++	++++
**Reference**	–	[S1,S2]	[S3]	[S4]	This manuscript

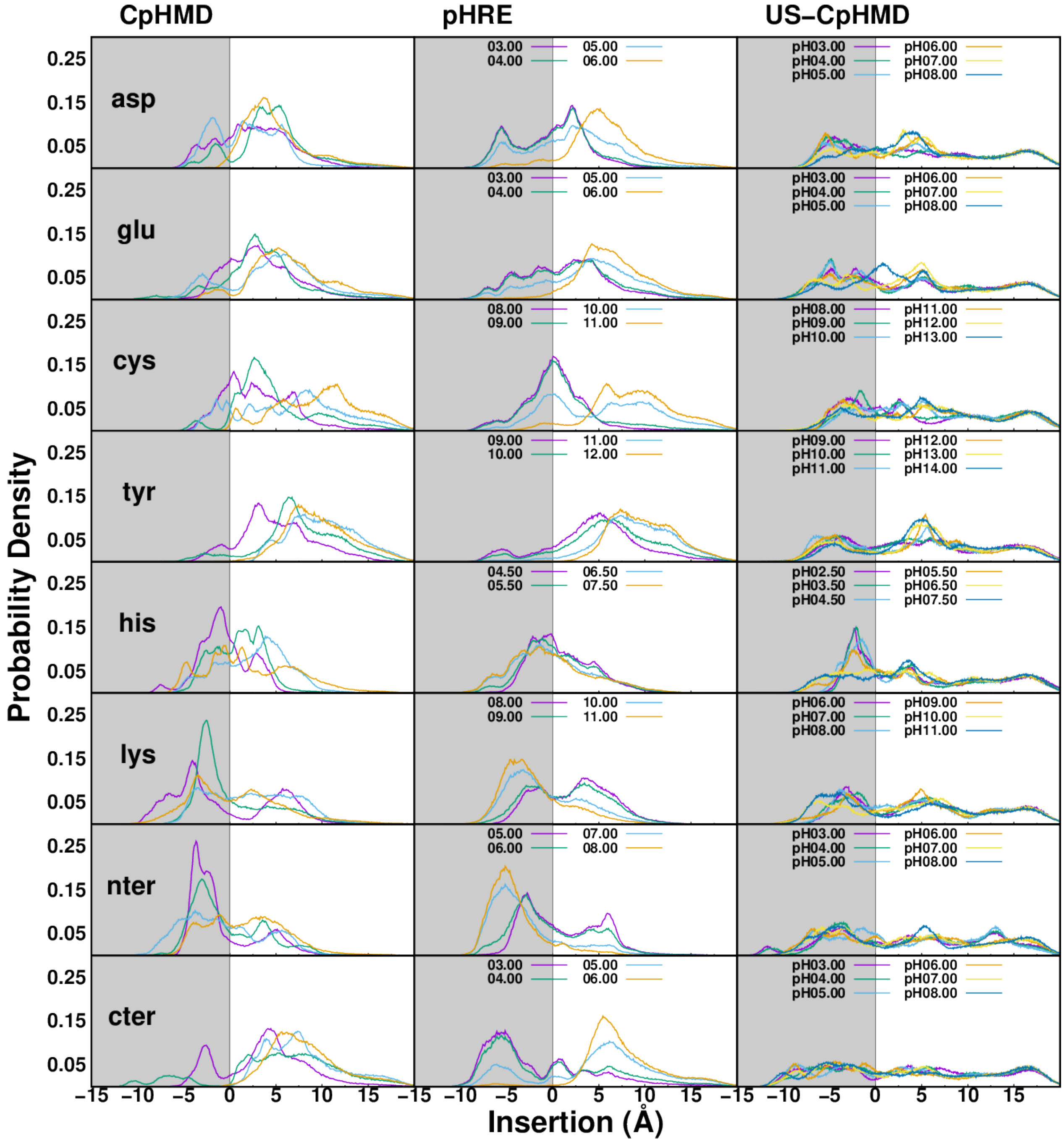

Figure S1: Insertion probability density calculated for all 8 pentapeptide systems, Asp, Glu, Cys, Tyr, His, Lys, NTer, and CTer, for all 100 ns triplicates at different pH values. Data for the three methods are shown, st-CpHMD, pHRE, and US-CpHMD from left to right.

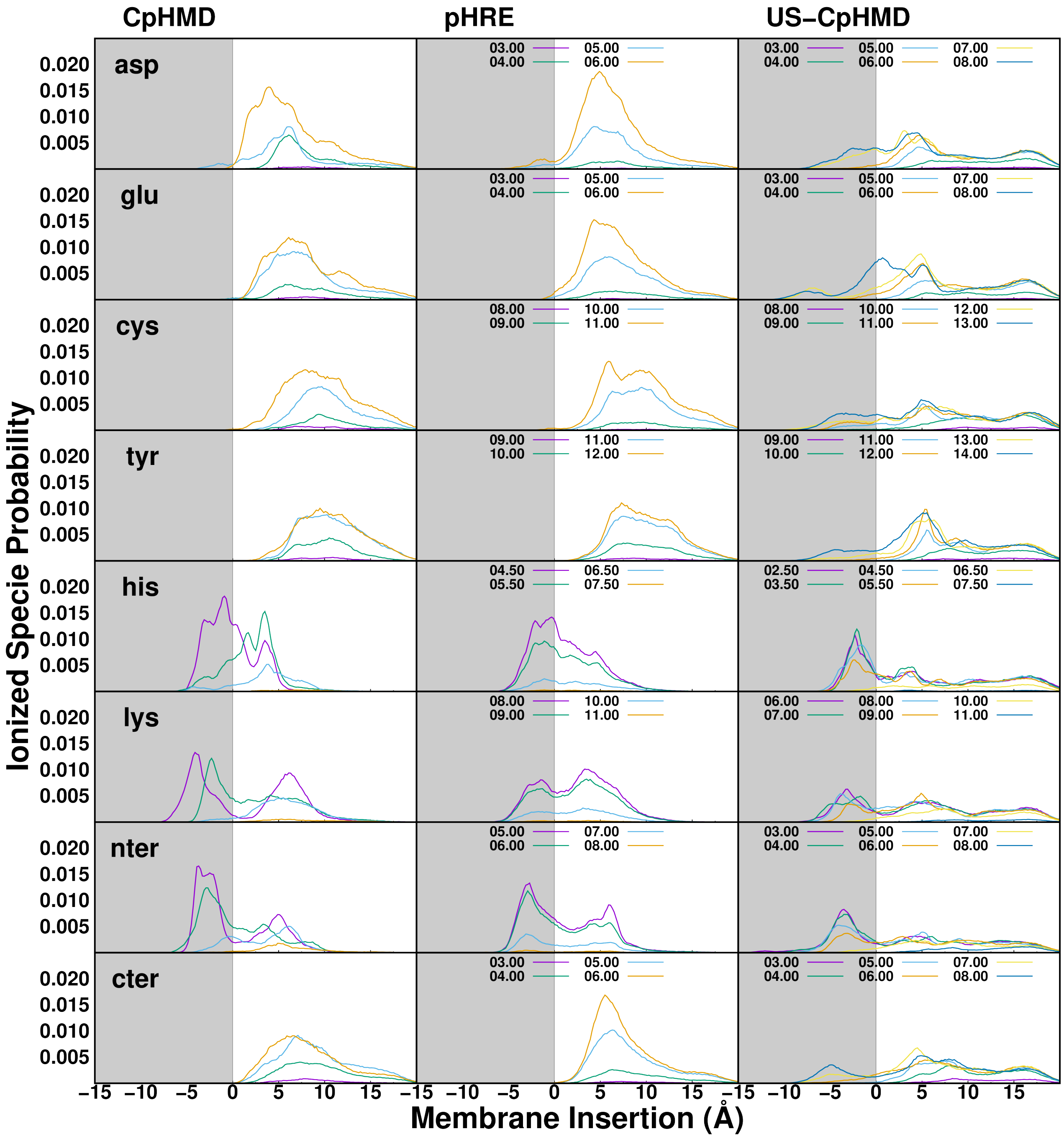

Figure S2: Probability of the ionized species along the membrane insertion axis, measured through MembIT, per pH value of each titratable group of the 8 pentapeptide systems, Asp, Glu, Cys, Tyr, Lys, His, N-ter, and C-ter. Populations were calculated using three distinct methods: CpHMD (left), pHRE (middle), and US-CpHMD (right; extended pH values). The region inside the membrane is shown as a gray area in the graph.

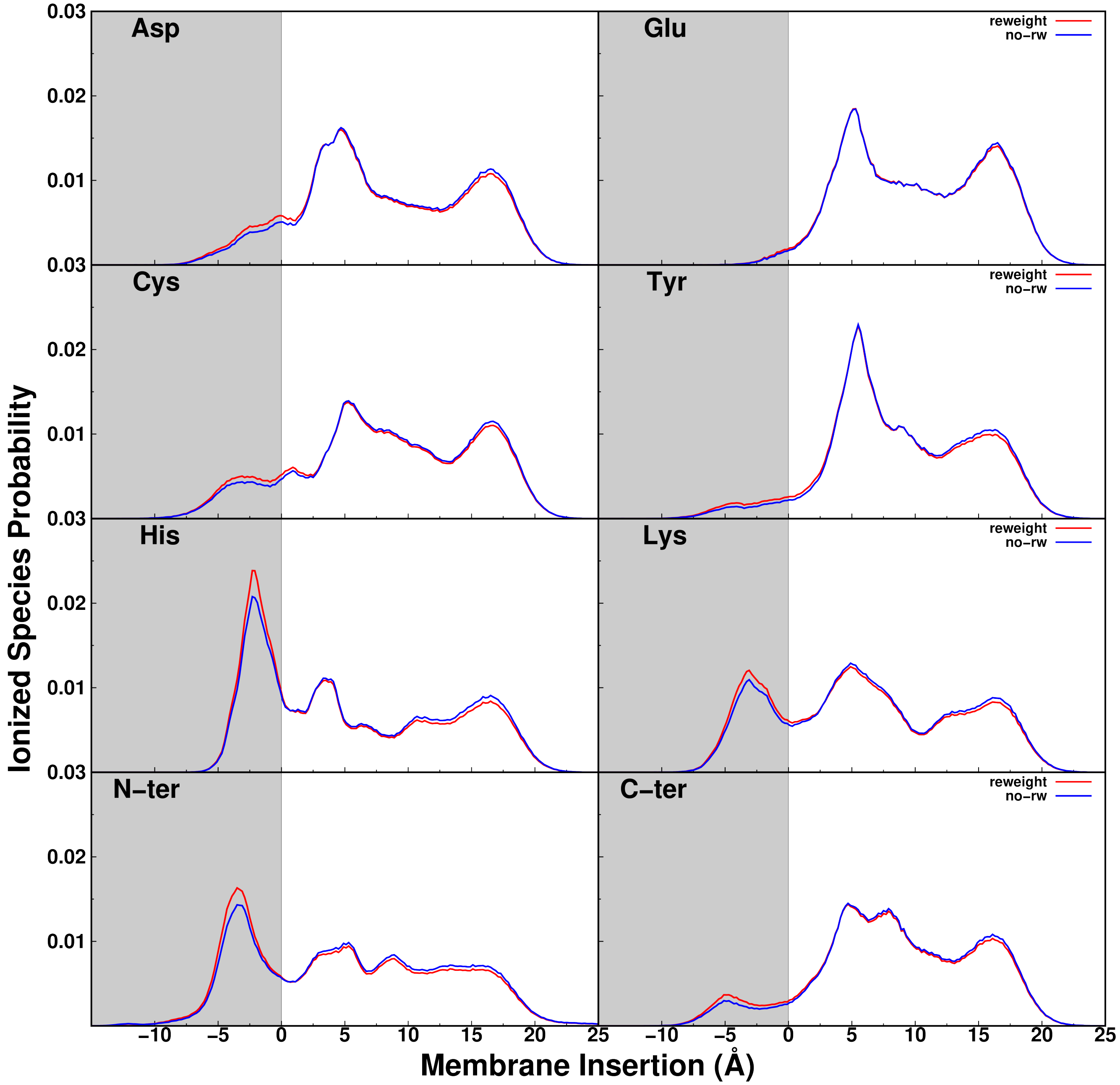

Figure S3: Probability of the ionized species along the membrane insertion axis in the US-CpHMD methodology for all 8 pentapeptides, Asp, Glu, Cys, Tyr, Lys, His, N-ter, and C-ter, with (red) and without re-weighting (blue). The region inside the membrane is shown as a gray area in the graph.

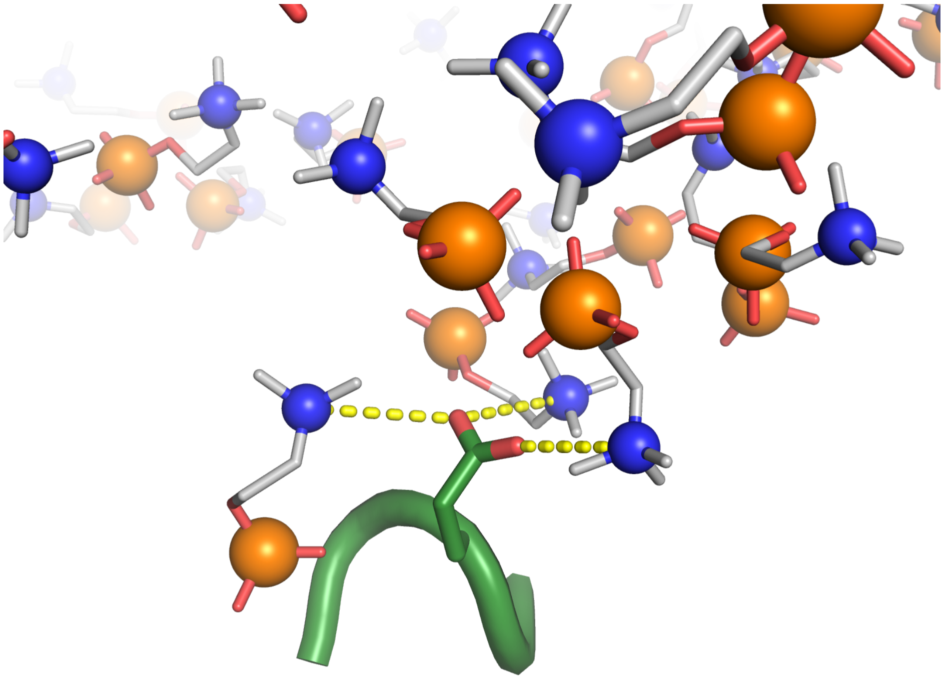

Figure S4: High energy conformation for Glu-containing pentapeptide at pH 8.0, showing a lipid choline group reoriented to stabilize the ionized carboxylate. The pentapeptide, depicted in green, is shown as a cartoon, with the Glu residue displayed as sticks. The lipid choline and phosphate groups are represented by gray sticks, with phosphorus and nitrogen atoms depicted as spheres.

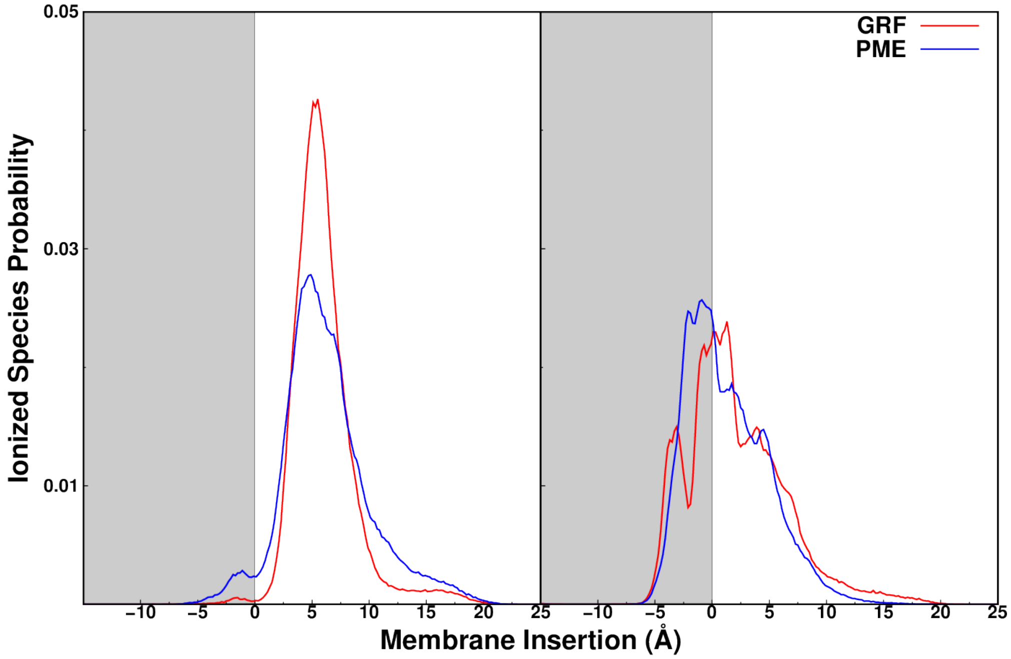

Figure S5: Anionic (Asp) and cationic (His) pentapeptide controls comparing between ionized specie probability in the 150 ns triplicate pHRE methodology simulations, using PME (blue) and GRF (red) as long-range electrostatic treatment.

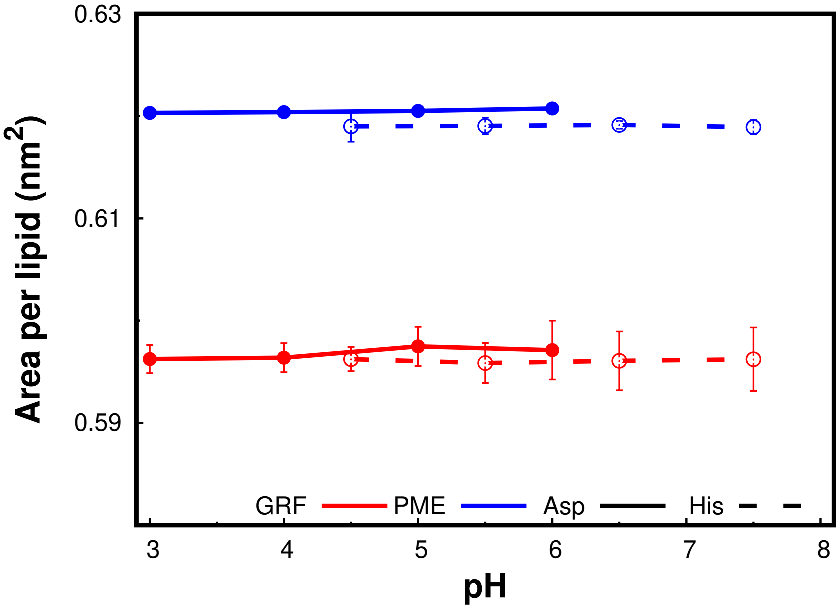

Figure S6: Area per lipid comparison between pHRE control triplicate simulations of an anionic (Asp - full line) and cationic (His - dashed line) pentapeptide system under PME (blue) and GRF (red) long-range electrostatic treatment. Errors were calculated using the standard error of the mean using the equilibrated portion of each replicate (last 75 ns).

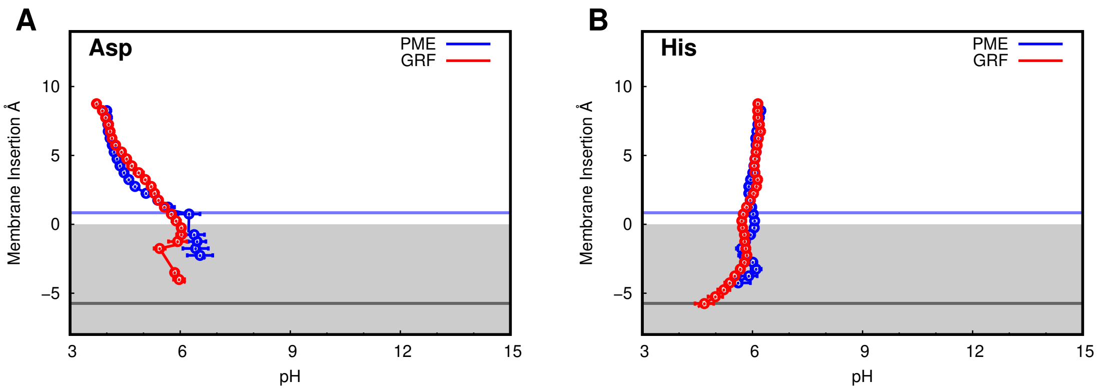

Figure S7: pK_a profile comparison between the anionic (Asp - A) and cationic (His - B) control pentapeptide systems treated with PME (blue) and GRF (red) in 3 replicates of 150 ns of pHRE simulation. The gray area represents the membrane region. A horizontal blue line indicates the average position of the choline nitrogen atoms at approximately 1 Å, and a dark gray horizontal line marks the start of the acyl chain region. Error bars were computed using the bootstrap method with 1000 bootstraps.

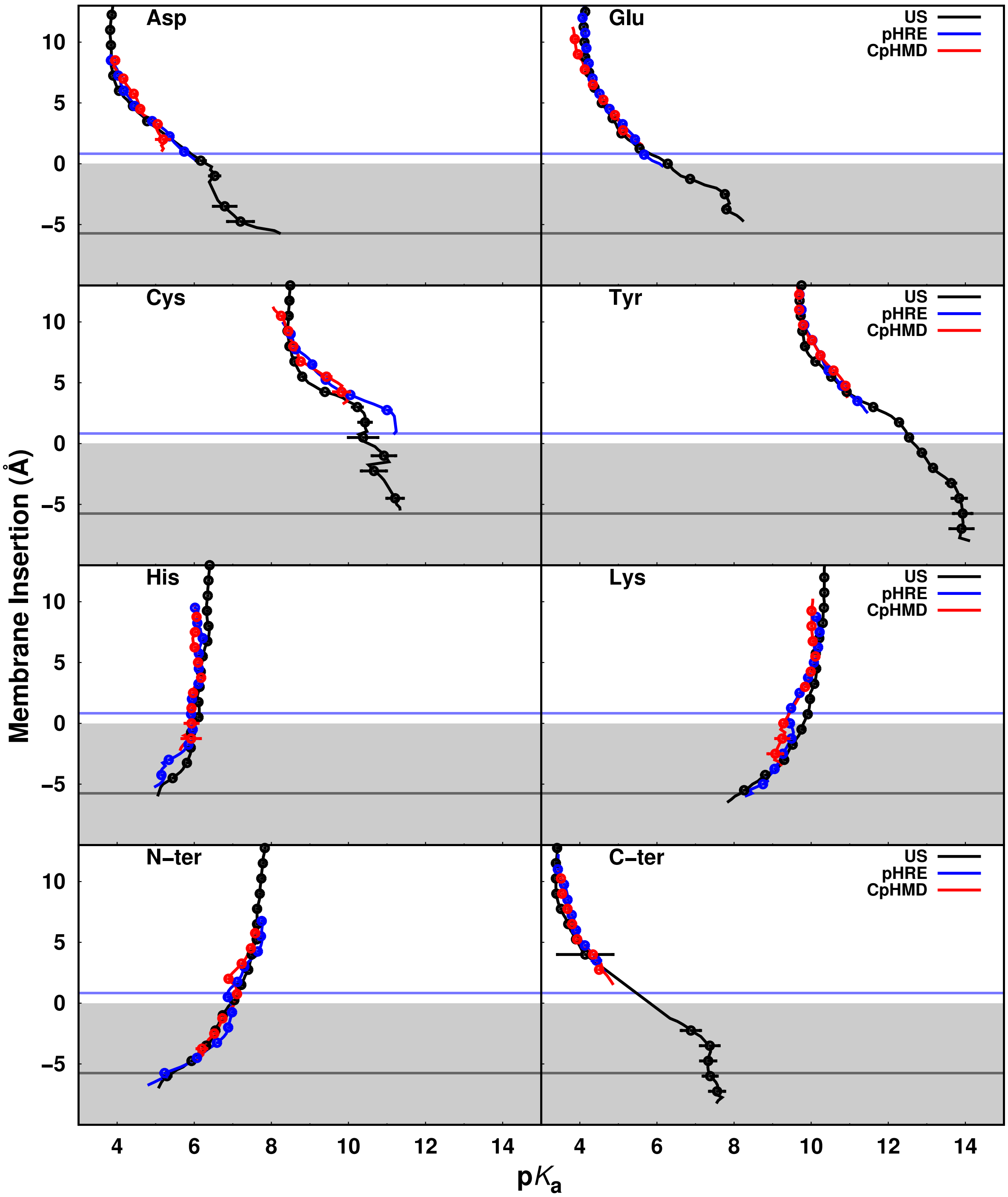

Figure S8: pK_a values along the membrane insertion coordinate of all pentapeptide systems. Three sampling methods are compared: CpHMD (red line), pH Replica-Exchange (pHRE; blue line), and US-CpHMD (black line). The gray area represents the membrane region. A horizontal blue line indicates the average position of the choline nitrogen atoms at approximately 1 Å, and a dark gray horizontal line marks the start of the acyl chain region. The pK_a values were calculated using the insertion obtained through the MembIT tool (see Methods). Error bars were computed using the bootstrap method with 1000 bootstraps.

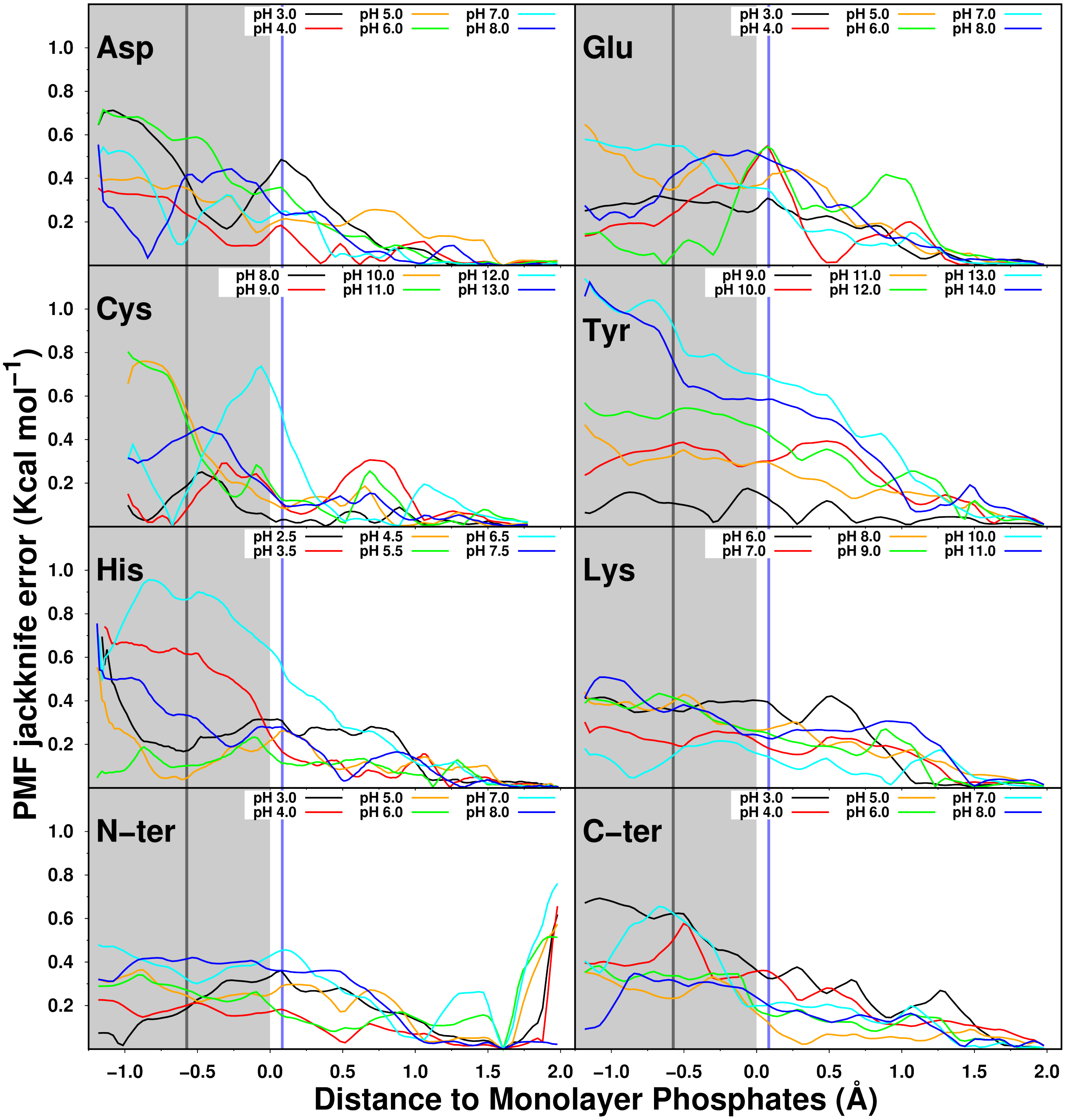

Figure S9: US-CpHMD obtained pH-dependent PMF profile errors for all amino acids and pH values. The error values were calculated using the jackknife leave-one-out strategy across different replicate datasets. The gray area represents the membrane region. A vertical blue line indicates the average position of the choline nitrogen atoms at approximately 1 Å, and the dark gray line marks the start of the acyl chain region.

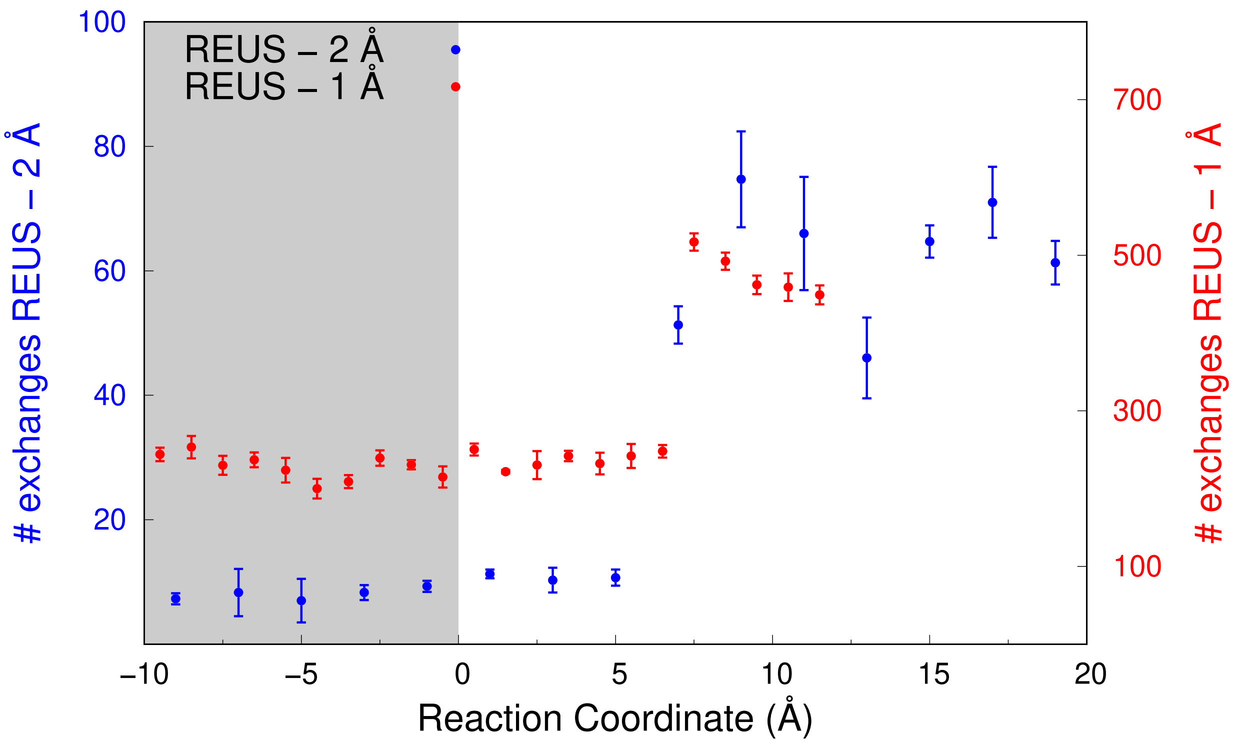

Figure S10: Number of umbrella windows exchanges accepted during REUS-CpHMD simulations of the 1 Å (red) and 2 Å (blue) US window separation. Since exchanges occur between adjacent US window pairs, the data is plotted at their midpoints. The gray-shaded area corresponds to the membrane-inserted regions.

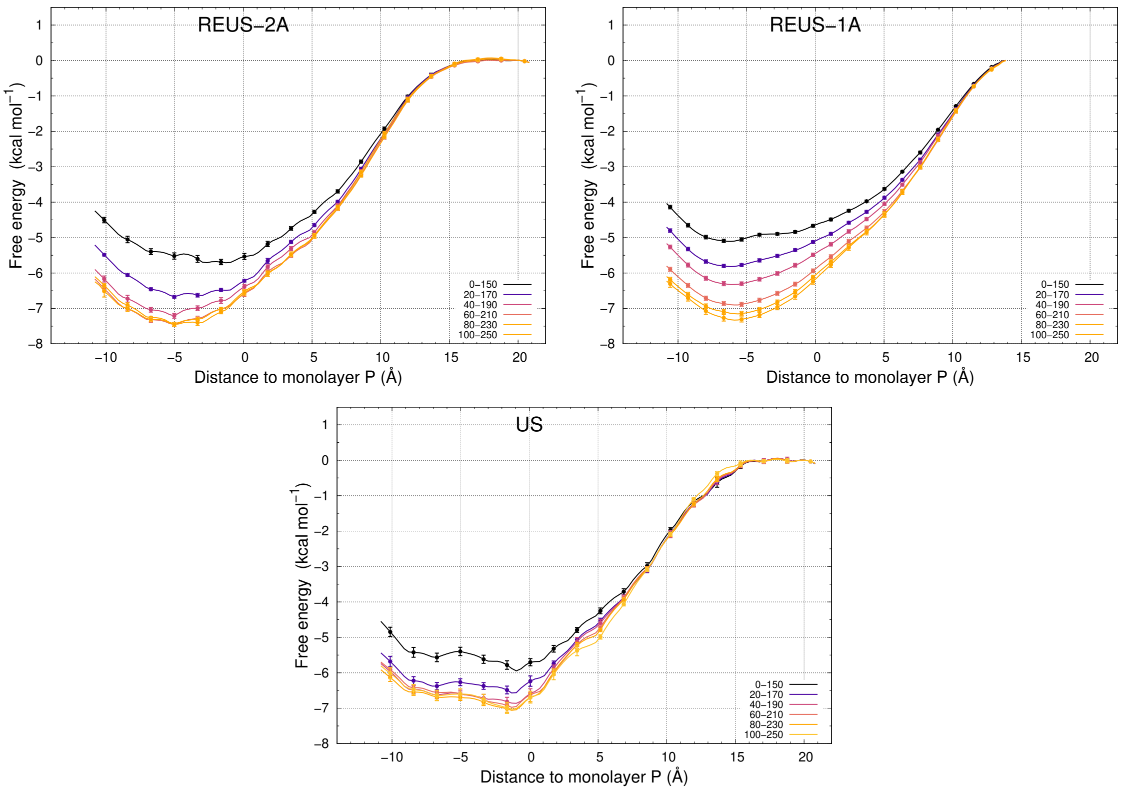

Figure S11: PMF profiles of US-CpHMD, REUS with US window separation of 2 Å, and REUS with US window separation at 1 Å. All profiles were obtained using the WHAM methodology, with a total simulation time of 150 ns at different starting points. Starting times were selected with a 20 ns step to evaluate convergence throughout the simulation.

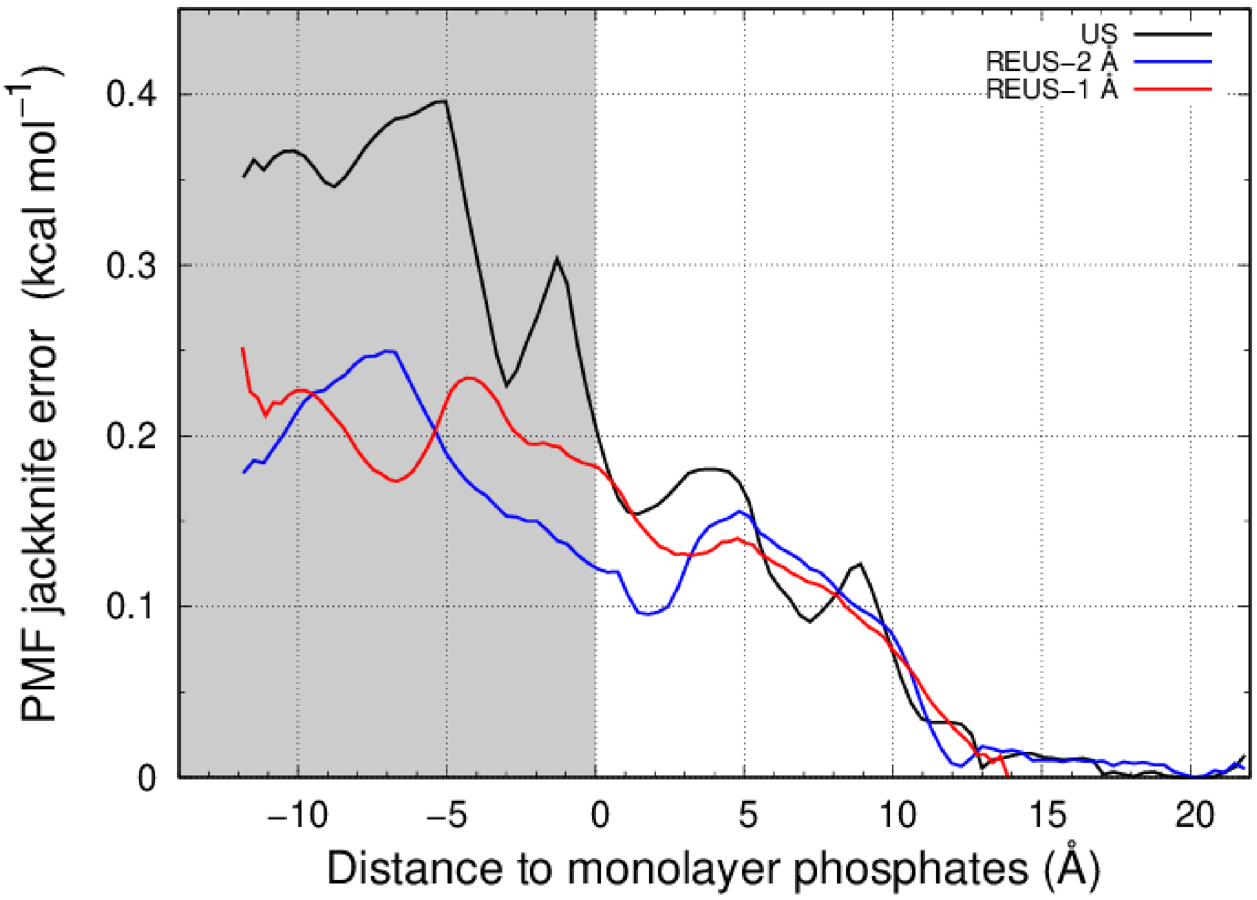

Figure S12: PMF profile jackknife errors of the US-CpHMD (black) and both REUS schemes, with US windows separated by 2 Å (blue) and 1 Å (red). The error values were calculated using the jackknife leave-one-out strategy across different replicate datasets. The gray area represents the membrane region.

References

(S1) Baptista, A. M.; Teixeira, V. H.; Soares, C. M. Constant-pH molecular dynamics using stochastic titration. *J. Chem. Phys.* **2002**, *117*, 4184–4200.

(S2) Machuqueiro, M.; Baptista, A. M. Constant-pH Molecular Dynamics with Ionic Strength Effects: Protonation–Conformation Coupling in Decalysine. *J. Phys. Chem. B* **2006**, *110*, 2927–2933.

(S3) Vila-Viçosa, D.; Reis, P. B.; Baptista, A. M.; Oostenbrink, C.; Machuqueiro, M. A pH replica exchange scheme in the stochastic titration constant-pH MD method. *J. Chem. Theory Comput.* **2019**, *15*, 3108–3116.

(S4) Oliveira, N. F.; Machuqueiro, M. Novel US-CpHMD Protocol to Study the Protonation-Dependent Mechanism of the ATP/ADP Carrier. *J. Chem. Inf. Model.* **2022**, *62*, 2550–2560.
